# Use of low dosage amino acid blends to prevent stress-related piglet diarrhea

**DOI:** 10.1093/tas/txab209

**Published:** 2021-10-26

**Authors:** Anna G Wessels, Tristan Chalvon-Demersey, Jürgen Zentek

**Affiliations:** 1 Institute of Animal Nutrition, Department of Veterinary Medicine, Freie Universität Berlin, Königin-Luise-Str. 49, 14195 Berlin, Germany; 2 METEX NOOVISTAGO, 32 rue Guersant, 75017 Paris, France

**Keywords:** arginine, branched-chain amino acids, cystine, pigs, stress, tryptophan

## Abstract

Weaning is a challenging period for piglets associated with reduced feed intake, impairment of gut integrity, and diarrhea. Previous studies demonstrate that supplementation with single functional amino acids (AA) promote piglets’ performance due to the improvement of intestinal health. Thus, we hypothesized that a combination of functional AA provided beyond the postulated requirement for growth could facilitate the weaning transition. Ninety piglets, initially stressed after weaning by 100 min overland transport, received a control diet or the same diet supplemented with a low-dosed (0.3%) mixture of AA (AAB-1: L-arginine, L-leucine, L-valine, L-isoleucine, L-cystine; AAB-2: L-arginine, L-leucine, L-valine, L-isoleucine, L-cystine, and L-tryptophan) for 28 days. Fecal consistency was ranked daily, growth performance was assessed weekly. On days 1 and 14 of the trial, blood samples were collected from a subset of 10 piglets per group to assess concentrations of insulin-like growth factor 1. After 28 days of feeding, tissues were obtained from the same piglets to analyze gut morphology and relative mRNA expression of genes related to gut function. Even if the stress response as indicated by rectal temperature was not different between the groups, pigs supplemented with AAB-2 showed firmer feces after weaning and less days with diarrhea compared to control. Furthermore, the jejunal expression of the MUC-2 gene was reduced (*P* < 0.05) in group AAB-2. Both AA mixtures increased crypt depth in the duodenum. Collectively, the given results indicate that 0.3% extra AA supplementation might alleviate postweaning diarrhea but did not alter growth performance of weanling piglets.

## INTRODUCTION

Early, abrupt weaning is a critical period for commercial piglets as they are exposed to stress associated with drastic changes in physiology, microbial colonization, and immune responses of the gastrointestinal tract (Campbell et al., 2013). Therefore, the first weeks after weaning are characterized by high prevalence of postweaning diarrhea and concomitant growth depression. Weaning can be characterized as multifactorial stress by accumulating environmental, dietary, and psychological stressors. Abrupt separation of piglets from their mother, mixing with other litters in a mostly new environment and after an unfamiliar transport causes in high level of social stress. The complete switch from highly digestible liquid milk to less digestible, more complex solid feed is another factor that contributes to reduced or completely discontinued feed intake (FI), which then leads to morphological changes, dysbiosis, and inflammation in the piglets’ intestines (Lallès et al., 2007). Those morphological changes are characterized by villus atrophy, crypt hyperplasia, and reduced production of mucus-producing goblet cells. As a result, there is a reduction in intestinal mucus production and an impairment of the gut barrier due to lesser tight junction expression. Nutrient transport is restricted (among others, by decreased enzyme activities), while pathogens and toxins can cross the intestinal barrier and cause an immune response (Lallès et al., 2007). This affects the animal welfare and the economic balance of the producers due to deteriorated growth performance and increased number of animal losses. The occurrence of piglet diarrhea therefore leads to the need for antibiotic treatment. The use of antibiotics in animals has raised concerns that the selective pressure on the bacteria population promotes antibiotic resistance. The European Union and many countries, including the United States, Canada, and Australia, have implemented measures and regulations to limit the use of antibiotics in animal agriculture. Nevertheless, the vast majority of total antibiotics administered in recent times were for prophylaxis, while metaphylaxis or treatment accounted for a much smaller proportion of total antibiotics (Lekagul et al., 2019). Eighty percent of antibiotics was administered to pigs <10 wk of age, and over 70% of the total indication in weaned pigs was due to gastrointestinal infections (Lekagul et al., 2019). For the sake of food safety and public health, it is reasonable to use nutritional feed additives to improve intestinal function of weaner pigs. The current experiment aimed to develop an effective strategy for optimized weaning diets by supplementing additional functional amino acids (AA). The AA focused on in the current study were arginine (Arg), the branched-chain AA (BCAA) leucine (Leu), valine (Val), and isoleucine (Ile), the sulfur AA cystine (Cys_2_), and tryptophan (Trp). The mixtures were designed based on following basic considerations: (1) Leu promotes protein synthesis but must be balanced to both other BCAA and Trp (Yin et al., 2010, Wessels et al., 2016a). Supplementation of BCAA enhances intestinal development and increases the production mucus in pigs (Mao et al., 2015; Sun et al., 2015). BCAA metabolites, such as isovalerate, isobutyrate, and 2-methylbutyrate, promote epithelial barrier function in vitro (Boudry et al., 2013). Tryptophan as precursor for the kynurenine pathway was shown to act anti-inflammatory in pigs suffering from experimental gut infection (Trevisi et al., 2009). (2) Cysteine supplementation in pigs regulates oxidative stress and reduces intestinal inflammation (Kim et al., 2009; Song et al., 2016). (3) In young pigs, Arg synthesis is inadequate for their optimal growth primarily because of the limited expression of N-acetylglutamate synthase in enterocytes (Zhang et al., 2014). Additional dietary Arg therefore promotes epithelial barrier function in pigs (Ewtushik et al., 2000). As recently hypothesized by Prates et al. (2021), we also based our experimental design on the hypothesis that AA have complementary modes of action and additional AA mixtures could help piglets cope with weaning in a healthy manner by maintaining gut health.

## MATERIAL AND METHODS

### Animals and Treatments

Experiments were conducted in accordance established guidelines for the care and handling of laboratory animals. All procedures involving handling and treatments of animals were approved by the local state office of occupational health and technical safety (Landesamt für Gesundheit und Soziales, Berlin, Germany, LaGeSo G 0183/18).

At the age of 28 days, 90 male and female piglets (Danbred × Piétrain) derived from a commercial site were weaned and immediately transported over 100 min duration as additional stress factor. Then, piglets were equally divided into three groups according to sex and body weight (BW) and randomly distributed to flat deck pens. Each pen housed a pair of one male and one female piglet. The nutrient levels and AA profile of the control diet (CTRL) were calculated to meet the requirements for weaned piglets (Gloaguen et al., 2013). The basal diet was supplemented with 0.3% of two different AA mixtures (AAB), AAB-1 containing 42% L-Arg, 33% BCAA, 25% L-Cys_2_; AAB-2 containing 31% L-Arg, 25% BCAA, 19% L-Cys_2_, and 25% L-Trp. The BCAA, L-Leu, L-Val, and L-Ile were premixed in a ratio of 2:1:1 as leucine acts as a nutrient signal to stimulate protein synthesis by initiating the mTOR pathway at cellular level but has to be provided in balance with Val and Ile (Wiltafsky et al., 2010; Wessels et al., 2016a,b). Feed ingredients and composition of the experimental diets are shown in Table 1. The piglets had ad libitum access to fresh drinking water and their respective experimental diet, which was offered in automatic feeders. Piglets individual BW, BW gain, and pen-wise FI were assessed weekly. A subset of 10 piglets per group was used to evaluate stress response by measuring rectal temperature at a time interval of 3 days each. This selection was made from the male piglets that had a weight at weaning close to the average weight of the whole population. The fecal consistency of the entire population was ranked daily by visual classification according to the scores 1 = liquid diarrhea, 2 = pasty feces falling out of shape upon contact with surfaces, 3 = formed feces, soft to cut, 4 = well-formed feces, firm to cut, 5 = hard and dry feces (obstipation). For this purpose, an average value of two piglets per pen was documented for each trial day. An average value <2 was evaluated as a day with diarrhea in the respective pen.

**Table 1.** Diets fed to weaner piglets during the 28-days experimental period (as-fed)

Treatment	CTRL	AAB-1	AAB-2
Ingredients, %			
Barley	25.000	25.000	25.000
Wheat	26.500	26.500	26.500
Maize	22.507	22.507	22.507
Soy	6.500	6.500	6.500
Milk powder	10.000	10.000	10.000
Rape oil	3.000	3.000	3.000
Calcium carbonate	1.106	1.106	1.106
MCP	1.089	1.089	1.089
Sodium bicarbonate	1.450	1.450	1.450
Sodium chloride	0.100	0.100	0.100
Mineral premix^1^	1.193	1.193	1.193
L-Lys HCl	0.670	0.670	0.670
L-Thr	0.300	0.300	0.300
DL-Met	0.220	0.220	0.220
L-Trp	0.105	0.105	0.105
L-Val	0.160	0.160	0.160
L-Ile	0.060	0.060	0.060
Econase XT	0.030	0.030	0.030
Phytase 0.85	0.010	0.010	0.010
Analyzed crude protein and amino acids, %			
Crude protein	16.52	16.23	16.42
Lys	1.25	1.27	1.26
Thr	0.80	0.82	0.81
Met + Cys	0.73	0.79	0.76
Trp	0.27	0.28	0.34
Ile	0.66	0.70	0.69
Val	0.88	0.92	0.90
Leu	1.24	1.28	1.27
Phe + Tyr	1.24	1.25	1.25
His	0.36	0.36	0.36
Arg	0.72	0.84	0.81
Calculated energy content, mineral concentrations, and SID amino acid ratios			
ME, MJ/ kg	13.58	13.64	13.65
NE, MJ/ kg	10.44	10.47	10.48
Ca, %	0.843	0.843	0.843
Available P, %	0.467	0.467	0.467
SID Lys, %	1.20	1.20	1.20
SID Thr:Lys	0.65	0.65	0.65
SID Met:Lys	0.40	0.40	0.40
SID Cys:Lys	0.19	0.25	0.23
SID (Met + Cys):Lys	0.60	0.66	0.65
SID Trp:Lys	0.22	0.22	0.28
SID Ile:Lys	0.53	0.54	0.54
SID Val:Lys	0.70	0.72	0.72
SID Leu:Lys	1.03	1.07	1.06
SID Phe:Lys	0.60	0.60	0.60
SID Tyr:Lys	0.41	0.41	0.41
SID (Phe + Tyr):Lys	1.01	1.01	1.01
SID His:Lys	0.30	0.30	0.30
SID Arg:Lys	0.58	0.68	0.66
SID Ala:Lys	0.48	0.48	0.48
SID Asp:Lys	0.83	0.83	0.83
SID Glu:Lys	2.74	2.74	2.74
SID Gly:Lys	0.39	0.39	0.39
SID Ser:Lys	0.55	0.55	0.55
SID Pro:Lys	1.10	1.10	1.10

CTRL, control group; AAB-1, control feed + 0.3% amino acid blend 1 (=0.125% arginine + 0.1% branched-chain amino acids + 0.075% cystine); AAB-2, control feed + 0.3% amino acid blend 2 (=0.094% arginine + 0.075% branched-chain amino acids + 0.056% cystine + 0.075% tryptophan); branched-chain amino acids = leucine:valine:isoleucine (2:1:1).

MCP, monocalcium phosphate; Lys, lysine; Thr, threonine; Met, methionine; Cys, cysteine; Trp, tryptophan; Ile, isoleucine; Val, valine; Leu, leucine; Phe, phenylalanine; Tyr, tyrosine; His, histidine; Arg, arginine; SID, standardized ileal digestible; Ala, alanine; Asp, aspartic acid; Glu, glutamic acid; Gly, glycine; Ser, serine; Pro, proline.

^1^Contents per kg premix: 400,000 IU Vit. A (acetate); 120,000 IU Vit. D_3_; 8,000 mg Vit. E (α-Tocopherol acetate); 200 mg Vit. K_3_ (MSB); 250 mg Vit. B_1_ (Mononitrate); 420 mg Vit. B_2_ (cryst. Riboflavin); 2,500 mg Niacin (Niacinamide); 400 mg Vit. B_6_ (HCL); 2,000 μg Vit. B_12_; 25,000 μg Biotin (commercial feed grade); 1000 mg pantothenic acid (Ca d-Pantothenate); 100 mg folic acid (commercial feed grade); 80,000 mg choline (chloride); 5,000 mg zinc sulfate; 5,000 mg iron carbonate; 6,000 mg manganese sulfate; 1,000 mg copper sulfate-pentahydrate; 20 mg sodium selenite; 45 mg calciumjodate; 130 g sodium chloride; 55 g Mg magnesium sulfate

### Sample Collection

On days 1 and day 14 after weaning, blood samples were collected from the *vena cava cranialis* of the subset 10 piglets per group after fasting overnight. Plasma was obtained by centrifugation (1,000 × *G* for 10 min at 4 °C) and was then stored at −80 °C. With the termination of the experiment on day 28, the same piglets were euthanized for sampling of intestinal tissues (duodenum, jejunum, and ileum), kidney, liver, spleen, and thymus. The tissues were weighted immediately and samples of the three gut sections were snap frozen in liquid nitrogen and stored at −80 °C until further analyses.

### Nitrogen and Amino Acid Quantification of the Diets

The nitrogen content of the diet was analyzed as standardized by the Association française de normalization (AFNOR, 1997). The AA content of the diets was analyzed by a JLC-500/V AminoTac Amino Acid Analyzer (Jeol, Croissy-sur-Seine, France) in the laboratory of METEX NØØVISTAGO (Amiens, France) according to a previously described method (AFNOR, 2005). To quantify methionine and cysteine, diet samples were oxidized with performic acid prior to hydrolysis. Amino acids were separated by ion exchange chromatography and measured by photometric detection after derivatization with ninhydrin. Total Trp was analyzed by HPLC after an alkaline hydrolysis with barium hydroxide (Commission Directive 2000/45/EC).

### Determination of Plasma Concentrations of IGF-1

Plasma concentrations of IGF-1were measured by a quantitative ELISA kit (Ref: MD58011, IBL International GmbH, Hamburg, Germany) according to manufacturer’s protocol. All concentrations were determined in duplicate.

### Quantitative PCR of Jejunal Samples

The jejunum, as the main site of action of the additional AA and as a section of the intestine with negligible microbial action, was chosen as the tissue for gene expression analysis. Total RNA was extracted using NucleoSpin RNA II kit (Macherey–Nagel GmbH and Company KG, Düren, Germany) according to the manufacturer’s instructions. The RNA quality and quantity were determined with the Agilent RNA 6000 Nano Kit in an Agilent 2100 Bioanalyzer (Agilent Technologies, Waldbronn, Germany). Transcription into cDNA was performed using the SuperScript III Reverse Transcriptase First-Strand complementary DNA Synthesis System (Invitrogen, Carlsbad, CA) in the AriaMx Real-Time PCR system (Agilent Technologies). The qPCR was performed using the Brilliant II SYBR Green QPCR Master Mix with Low ROX (Agilent Technologies) on a Stratagene MX3000p (Agilent Technologies). The expression of genes related to barrier function (Le Floc’h et al., 2018) was assessed. Namely, zona occludens-1, claudin-1, claudin-2, claudin-4, and occludin as well as Mucin 2 (MUC2). Several studies have demonstrated that both ornithine decarboxylase and diamine activity rise during adaptive hyperplasia (Wolvekamp and de Bruin, 1994) as it occurs with weaning (Hayakawa et al., 2016). The 60S ribosomal protein L13 (RPL13), succinate dehydrogenase subunit A (SDHA), and β2-microglobulin (β2-glob) were selected as reference genes. PCR efficiency correction and normalization using the reference genes mentioned were performed according to Pfaffl et al. (2002). Primer sequences and annealing temperatures of the assessed genes were taken from internal standards.

### Histomorphology of Duodenum, Jejunum, and Ileum Samples

Tissue sections from duodenum, jejunum, and ileum were opened longitudinally, placed on cork boards with hedgehog spines and fixed in a 4% phosphate-buffered formaldehyde solution for 24 h. After dehydration and infiltration with solidified paraffin wax, the samples were embedded. The paraffin blocks were cut at 4 μm with a sledge microtome (SM 2000 R, Leica, Nussloch, Germany). Obtained sections were mounted on glass slides. Tissue slides were stained with Haematoxylin-Eosin and analyzed with a light microscope (Photomicroscope III, Zeiss, Oberkochen, Germany) equipped with a digital camera (DP72, Olympus, Tokyo, Japan). Histomorphometric parameters were measured using an image analysis software (CellSense Software, Olympus, Tokyo, Japan). In total, 15 vertically oriented villi and crypts per section were analyzed. The villus length (measured from the tip of the villi to the villus crypt junction) and crypt depth (defined as the depth of the invagination between adjacent villi) was measured and the villus length-to-crypt depth ratio calculated.

### Statistical Analyses

The statistical analyses were performed using the software package SPSS (IBM SPSS Version 25). Prior to one-way ANOVA based on treatment number, zootechnical, and physiological data were tested for normal distribution by Shapiro–Wilk test and variance homogeneity by Levene’s test. Potential covariates (sex, BW at weaning, and room) were considered and included to the statistical model in case of significant impact on single target parameters. All treatment means were compared with each other using Tukey test or Games–Howell test depending on variance homogeneity. As a rank variable, fecal score per day was analyzed using the Kruskal–Wallis test. The number of days with diarrhea (fecal score <2) as a metric variable was analyzed by ANOVA and Tukey test. The statistical tests used are shown in the footnotes of the respective data tables. Mean differences with a probability of *P* < 0.05 were accepted as statistically significant, also mean differences with *P*-values ranging from 0.06 to 0.10 were considered as trends. The gene expression data of jejunal tissue were analyzed by REST 2009 software (Qiagen GmbH, Munich, Germany; Pfaffl et al., 2002). In the REST, gene expression analysis, the treatment groups AAB-1, and AAB-2 were compared with the CTRL group after PCR efficiency correction and normalization by reference genes (RPL13 and SDHA).

## RESULTS

### Zootechnical Performance, Rectal Temperature, and Incidence of Diarrhea

**Figure 1.**
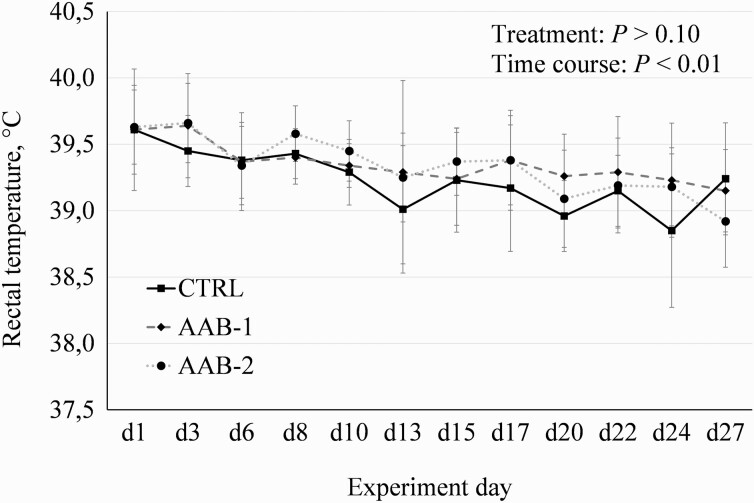
Development of the rectal temperature postchallenge of piglets fed either a control feed or experimental feed supplemented with AA mixtures. CTRL, control group; AAB-1, control feed + 0.3% amino acid blend 1 (=0.125% arginine + 0.1% branched-chain amino acids + 0.075% cystine); AAB-2, control feed + 0.3% amino acid blend 2 (=0.094% arginine + 0.075% branched-chain amino acids + 0.056% cystine + 0.075% tryptophan); branched-chain amino acids = leucine:valine:isoleucine (2:1:1); Weaning and additional transport challenge resulted in higher rectal temperatures at the onset of the experiment with linear decrease over time (*P* < 0.01). Rectal temperatures did not differ among the groups (*n* = 10).

Dietary supplementation with 0.3% AAB-2 reduced (*P* < 0.05) the number of days with diarrhea by 59% in comparison with CTRL (Table 2). Weaning and additional transport challenge resulted in higher rectal temperatures at the onset of the experiment with linear decrease over time (*P* < 0.01). However, rectal temperatures were not different among the groups (Fig. 1). From days 21 to 28 of the experiment, gain-to-feed ratio (G:F; *P* > 0.05) was significantly reduced in AAB-1, accompanied by a trend for elevated average daily FI (*P* < 0.10). Apart from this, no significant differences were observed in the performance of the experimental groups (Table 3).

**Table 2.** Average fecal scores and count of days with diarrhea of weaner piglets during the 28-days experimental period^1^

Treatment	CTRL	AAB-1	AAB-2	SEM	P-value
*Fecal scores, range from 1 (liquid diarrhea) to 5 (obstipation)*					
Day 5	2.79	3.47	3.29	0.091	0.098
Day 6	2.86	3.33	3.21	0.084	0.097
Day 7^2^	2.79^a^	2.70^a^	3.36^b^	0.088	0.012
Day 10	2.81	2.87	3.29	0.093	0.091
Day 22^2^	3.00^ab^	2.87^a^	3.18^b^	0.064	0.024
Day 23^2^	2.79^a^	2.87^a^	3.29^b^	0.081	0.021
Average score (days 1 to 28)	3.01	3.05	3.19	0.033	0.084
*Number of days with diarrhea (score < 2)*					
Score < 2^3^	3,29^b^	2,54^ab^	1,36^a^	0.261	0.033

CTRL, control group; AAB-1, control feed + 0.3% amino acid blend 1 (=0.125% arginine + 0.1% branched-chain amino acids + 0.075% cystine); AAB-2, control feed + 0.3% amino acid blend 2 (=0.094% arginine + 0.075% branched-chain amino acids + 0.056% cystine + 0.075% tryptophan); branched-chain amino acids = leucine:valine:isoleucine (2:1:1).

^1^Data are presented as means (*n* = 15).

^a,b^Values within a row with different superscripts differ significantly at *P* ≤ 0.05.

^2^Kruskal–Wallis for rank variable.

^3^Tukey test for metric variable.

**Table 3.** Zootechnical performance of weaner piglets during the 28-days experimental period^1^

Treatment	CTRL	AAB-1	AAB-2	SEM	P-value
*Body weight, kg*					
IBW	7.8	7.9	7.8	0.24	0.956
Day 7	8.6	8.8	8.5	0.21	0.818
Day 14	10.0	10.2	9.9	0.20	0.736
Day 21	12.1	12.9	12.2	0.31	0.437
Day 28	16.1	16.4	15.6	0.38	0.653
*Daily weight gain, g/d*					
Day 7	107	129	105	8.3	0.428
Day 14	187	187	195	8.0	0.893
Day 21	341	384	330	16.0	0.331
Day 28	498	492	485	20.3	0.963
Days 1 to 28	285	290	279	10.1	0.913
*Daily feed intake, g/d*					
Days 1 to 7	160	183	156	10.2	0.471
Days 7 to 14	328	353	312	15.4	0.562
Days 14 to -21	519	575	481	20.7	0.199
Days 21 to 28	779	853	710	35.9	0.087
Days 1 to 28	448	491	423	16.4	0.223
*Gain-to-feed ratio, kg:kg*					
Days 1 to 7	0.69	0.73	0.64	0.022	0.380
Days 7 to 14	0.57	0.61	0.67	0.021	0.214
Days 14 to 21	0.65	0.68	0.70	0.019	0.594
Days 21 to 28	0.63^ab^	0.58^a^	0.65^b^	0.012	0.031
Days 1 to 28	0.64	0.62	0.66	0.011	0.193

CTRL, control group; AAB-1, control feed + 0.3% amino acid blend 1 (= 0.125% arginine + 0.1% branched-chain amino acids + 0.075% cystine); AAB-2, control feed + 0.3% amino acid blend 2 (= 0.094% arginine + 0.075% branched-chain amino acids + 0.056% cystine + 0.075% tryptophan); branched-chain amino acids = leucine:valine:isoleucine (2:1:1).

IBW, initial body weight; BW, body weight at day *x* of the experiment; dWG, daily weight gain; dFI, daily feed intake; FCR, feed conversion ratio.

^1^Data are presented as means (*n* = 15).

^a,b^Values within a row with different superscripts differ significantly at *P* ≤ 0.05 (Games–Howell test).

### Plasma Concentrations of IGF-1 and Tissue Weights

No significant differences were observed in plasma concentrations of IGF-1 between CTRL and AAB groups (Table 4). No differences were observed among the groups concerning tissue weights at day 28 after weaning (*P* > 0.05).

**Table 4.** Plasma concentrations of insulin-like growth factor 1 (IGF-1)^1^ on days 1 and 14 of the experiment

Treatment	CTRL	AAB-1	AAB-2	SEM	*P*-value
IGF-1 day 1, ng/mL	80.5	63.2	94.0	5.52	0.059
IGF-1 day 14, ng/mL	93.2	102.1	116.1	6.18	0.325
Difference, ng/mL	15.8	31.01	22.3	8.53	0.807

CTRL, control group; AAB-1, control feed + 0.3% amino acid blend 1 (=0.125% arginine + 0.1% branched-chain amino acids + 0.075% cystine); AAB-2, control feed + 0.3% amino acid blend 2 (=0.094% arginine + 0.075% branched-chain amino acids + 0.056% cystine + 0.075% tryptophan); branched-chain amino acids = leucine:valine:isoleucine (2:1:1);

^1^Data are presented as means (*n* = 10).

### Intestinal Histomorphology and Gene Expression in Jejunal Tissue

As shown in Table 5, dietary supplementation of both AAB increased (*P* < 0.05) crypt depth of the duodenum of piglets, in comparison with CTRL (*P* < 0.05). Supplementation of AAB-2 reduced the mRNA levels for MUC2 in the jejunum of piglets, as compared with the control group (*P* < 0.05; Table 6).

**Table 5.** Intestinal morphology parameters of three gut segments^1^

Treatment	CTRL	AAB-1	AAB-2	SEM	*P*-value
*Duodenum, µm*					
Villus length	565	564	560	24.1	0.996
Crypt depth	261^a^	299^b^	299^b^	5.9	0.009
V:C ratio	2.26	1.90	1.88	0.092	0.212
*Jejunum, µm*					
Villus length	514	450	482	21.3	0.480
Crypt depth	215	219	204	5.3	0.506
V:C ratio	2.43	1.99	2.29	0.121	0.294
*Ileum, µm*					
Villus length	355	338	346	12.2	0.851
Crypt depth	209	208	203	4.8	0.888
V:C ratio	1.73	1.63	1.74	0.074	0.759

CTRL, control group; AAB-1, control feed + 0.3% amino acid blend 1 (=0.125% arginine + 0.1% branched-chain amino acids + 0.075% cystine); AAB-2, control feed + 0.3% amino acid blend 2 (=0.094% arginine + 0.075% branched-chain amino acids + 0.056% cystine + 0.075% tryptophan); branched-chain amino acids = leucine:valine:isoleucine (2:1:1).

VH = villus height, CD = crypt depth.

^1^Data are presented as means (*n* = 10).

^a,b^Values within a row with different superscripts differ significantly at *P* ≤0.05 (Tukey test).

**Table 6.** Relative expression of jejunal genes related to gut barrier function, gut maturation, or immune response^1^

Treatment	CTRL	AAB-1	AAB-2	*P*-value
*Gut barrier*				
Claudin-1	1.000	1.232	0.834	>0.05
Claudin-2	1.000	0.888	0.875	>0.05
Claudin-4	1.000	1.090	0.776	>0.05
Mucin 2	1.000^b^	0.824^b^	0.693^a^	<0.01
Occludin	1.000	1.057	1.021	>0.05
Zona occludens-1	1.000	1.283	0.969	>0.05
*Gut maturation*				
Ornithine decarboxylase	1.000	1.074	0.978	> 0.05
*Immune response*				
Diamine oxidase	1.000	0.969	0.886	> 0.05

CTRL, control group; AAB-1, control feed + 0.3% amino acid blend 1 (=0.125% arginine + 0.1% branched-chain amino acids + 0.075% cystine); AAB-2, control feed + 0.3% amino acid blend 2 (=0.094% arginine + 0.075% branched-chain amino acids + 0.056% cystine + 0.075% tryptophan); branched-chain amino acids = leucine:valine:isoleucine (2:1:1).

^1^Data are expressed as relative values in relation to the control group (*n* = 10).

^a,b^Values within a row with different superscripts differ significantly at *P* ≤ 0.05 (REST 2009); reference genes: 60S ribosomal protein L13 (RPL13), succinate dehydrogenase subunit A (SDHA) and β2-microglobulin (β2-glob).

## DISCUSSION

During critical metabolic conditions, such as weaning piglets, both indispensable, and dispensable AA are nowadays known to be inadequately supplied for maximal growth and optimal health when FI is low, especially with the provision of protein-reduced diets. The present study was conducted to investigate the effects of two low-dosed AA mixtures, consisting of L-Arg, L-Leu, L-Val, L-Ile, L-Cys_2_, and in AAB-2 group additionally L-Trp, on the growth performance and intestinal properties of weaner pigs. Our results showed that dietary supplementation with 0.3% AAB had no significant effect on growth performance. However, AAB-2 markedly reduced the frequency of diarrhea as indicated by days with an average fecal score <2 accompanied with a reduced gene expression of jejunal MUC2. The reduced frequency of diarrhea may indicate an improvement of intestinal health, which could be explained by synergistical effects of the single AA components even if provided in low dosage. All extra supplemented AA used in the current experiment were selected based on their health promoting effects. These include important physiological mechanisms such as protection against oxidative stress and inflammation, activation of mTOR in intestinal tissues, modulation of intestinal inflammatory responses, and attenuation of villus atrophy (Le Floc’h et al., 2018).

To our knowledge, this is one of the pioneer studies published using such complex AA mixtures consisting of five to six single AA. Growth stimulating effects were described in experiments supplementing 0.6% to 1.0% Arg (Wu et al., 2010; Yao et al., 2011; Wang et al., 2012), 0.54% BCAA blend (Zhang et al., 2013), >0.25% L-cysteine (Song et al., 2016), 0.05% N-acetylcysteine to piglets challenged with lipopolysaccharide (LPS; Hou et al. ,2012) or different levels of L-Trp (Fernández et al., 2009; Trevisi et al., 2009; Li et al., 2016). This is not confirmed by other studies without significant performance enhancements from supplementation of L-Arg (Corl et al., 2008; Liu et al., 2008; Zhan et al., 2008; Bergeron et al., 2014) or L-Trp (Koopmanns et al., 2006; Tossou et al., 2016; Jayaraman et al., 2017). All studies named used higher doses of the single AA. The present study was designed to test whether a low dose of individual AA in combination already showed efficiency, but the supplements had no effects on either growth performance or organ weights. Since the diarrhea frequency in the present study was low anyway, the reduction of days with diarrhea by half in the AAB-2 group did not lead to an improvement in growth performance. Since our control diet was designed to cover weaners’ requirement for AA, the question arises whether stronger effects would be more pronounced in a longer-lasting challenge model, such as hygienic deficiencies or experimental infections. However, no effects on performance were recorded by Prates et al (2021) with comparable AA combinations even under LPS challenge. An important index in the evaluation of stress in domestic animals is rectal temperature (Hamilton et al., 2004; Ritter et al., 2009), since endogenous pyrogens are released in response to bacteria and their toxins, immunological and inflammatory diseases (Zampronio et al., 1994), and to stress (Ritter et al., 2009). The data on rectal temperature show such a thermal stress response to weaning and subsequent transport. However, since the average rectal temperatures were higher than reference values for piglets of this age (Heinritzi et al, 2006) only at the first two measurements, it must be acknowledged in the present experiment that the challenge was of relatively short duration compared with experimental infections or hygiene challenges. It was shown recently that unhygienic reared animals use more nutrients to the adaptation to the challenging conditions (Van der Meer et al., 2016), since an immune stimulation changes animal’s nutrient requirements, particularly for AA. Recent studies with LPS or *Escherichia coli* challenge have shown indeed that the requirements for methionine and cysteine (Rakhshandeh et al., 2010), threonine (Ren et al., 2014), and Trp (Koopmans et al., 2006, 2012) are increased in case of immune system stimulation.

To evaluate the dietary potential, particularly that of BCAA supplementation, on endocrine growth factors, plasma IGF-1 was analyzed. IGF-1 is described as a mediator of the anabolic and mitogenic activity of growth hormone (Laron, 1999). Both IGF-1 and Leu stimulate skeletal muscle growth, which is related to the activation and proliferation of myogenic satellite cells in skeletal muscle of young pigs (Han et al., 2008). However, supplementation of AA in the present study had no effect on IGF-1 levels. Although there is widespread agreement that blood concentration of IGF-I is positively correlated with growth rate in pigs (*r*_g_ = 0.15; Buonomo et al., 1987). In contrast, however, pig breeding associations aim for low juvenile IGF-1, which is subject to considerable genetic influence with a mean heritability (*h*^2^ = 0.24). Juvenile IGF-1 exhibits a strong positive genetic correlation with backfat thickness (*r*_g_ = 0.46). This genetic correlation is accounted for in pig breeding by a down selection strategy for juvenile IGF-1 (Hermesch et al., 2001). Further, it was demonstrated that the process of weaning reduces the concentrations of blood IGF-1 significantly (Carroll et al., 1998, Matteri et al., 2000), likely caused by the weaning associated reduction in FI. Due to the genetic impact, the likelihood of genetic intervention, and the impact of weaning the use of blood IGF-1 as marker for successful nutritional intervention might be revised. This assumption is underlined by a low effect size (ƒ = 0.29) of the dietary treatments on IGF-1 calculated in the current experiment, which indicates that differences were caused by parental effects and weaning (adaptation) more than by dietary treatment. However, Wang et al. (2012) found an increase in jejunal IGF-1 concentrations in IUGR piglets treated with 0.6% L-Arg to a milk replacer diet. In contrast, as in the present study, Whang et al. (2003) and Cho et al. (2008) detected no differences in serum concentrations of IGF-1 and growth performance of pigs fed different levels of dietary crude protein.

The integrity of the digestive tract can be compromised by interdependent factors such as stress (e.g., weaning and transport), lack of FI, abrupt dietary changes, dysbiosis, and inflammation. As a result, the digestive capacity and the function of the mucosal barrier are deteriorated. Dysregulations of the intestinal mucosal barrier, as well as the invasion of pathogenic organisms, are associated with disease (Le Floc’h et al., 2018). As a result, early weaned pigs often experience diarrhea caused by impaired mucosal barrier function (Campbell et al., 2013; Wang et al., 2015). An increase in intestinal permeability is associated with villous atrophy, crypt hyperplasia, and a significant decrease in jejunal expression of TJ proteins. Abrupt weaning results in a dramatic reduction in villus height and a highly significant increase in crypt height in the small intestine (Hampson, 1986), indicating the negative effects of acute weaning stress on intestinal maturation. Deeper crypts indicate rapid enterocyte turnover (Chen et al., 2019). In this study, duodenal crypt depth significantly was increased in both treatment groups whereas no effect was detected on villus height or the ratio of villus height-to-crypt depth. Enterocytes of the small intestine account for up to 90% of all cells within the crypts, and an even larger proportion of cells are found in the villi determining absorption and digestion (Chen et al., 2019). Therefore, small intestine epithelial cells may be a biomarker to reflect intestinal function, which is affected by diet, weaning stress, and pathogens in the environment (Yan et al., 2018). However, it stands to reason that weaning and transport stress was no longer evident at the physiological level at the time of dissection 28 days after the initial stressors of the current experiment. The hypothesis is supported by the decline in rectal temperature and the fact that TJ proteins in the present study did not show any changes as a function of feeding. The relative expression of MUC-2, an important defense protein of the intestinal layer, was reduced in the ABB-2 group compared with the control group. This finding could explain the lower frequency of diarrhea in treatment group AAB-2. Inflammatory symptoms in intestinal diseases caused by the proliferation of intestinal pathogens, in particular, lead to increased secretion from the intestinal mucosa (Ayoola et al., 2015). Therefore, the decreased MUC2 level possibly indicates a lower stimulation of the intestinal immune response due to a less aggressive intestinal microbiota.

In summary, the present experiment revealed no significant effects on performance and physiological parameters of weanling piglets by low dose supplementation of 0.3% AA mixtures consisting of L-Arg, BCAA, L-Cys_2_ or L-Arg, BCAA, L-Cys_2_, and L-Trp. However, the AA supplementation including L-Trp had a positive effect on the fecal consistency during the 4 wk of weaning. Future research based on the given results could thus contribute to improved piglet health and a reduction in therapeutic antibiotic use in pig farming in the long term.
